# Determining the prognostic value of CRP and neutrophil lymphocyte ratio in patients hospitalized for deep neck infection

**DOI:** 10.1016/j.bjorl.2024.101492

**Published:** 2024-08-10

**Authors:** Recep Haydar Koç, Mehmet Akif ABakay, İbrahim Sayın

**Affiliations:** a Sultangazi Haseki Training and Research Hospital, Department of Otorhinolaryngology and Head and Neck Surgery, Istanbul, Turkey; b Bakırköy Dr. Sadi Konuk Training and Research Hospital, Department of Otorhinolaryngology and Head and Neck Surgery, Istanbul, Turkey

**Keywords:** Neck, Infection, Abscess, C-reactive protein (CRP), Neutrophil

## Abstract

**Objectives:**

This study aims to assess the impact of the Neutrophil/Lymphocyte Ratio (NLR) and C-Reactive Protein (CRP), both markers of systemic inflammation, on the duration of hospitalization for Deep Neck Infections (DNIs).

**Methods:**

The research encompassed patients of all age groups admitted between January 2016 and January 2021 due to DNIs. Patient data, including demographic details, etiology, comorbidities, radiological findings, treatment specifics, laboratory results (CRP values, leukocyte counts, neutrophil counts, lymphocyte counts, NLR), culture outcomes, length of stay, complications, mortalities were retrospectively evaluated. Patients were categorized into two groups based on their hospitalization duration: less than 7-days and 7-days or more. Univariate and multivariate analyses were conducted to examine the association between age, NLR, CRP, and hospital stay length.

**Results:**

The study encompassed 275 patients, with a mean age of 36 ± 20.2 years. The mean hospital stay was 9.6 ± 6.6 days. Tonsillopharyngeal infections were the most common etiology (34%). Notably, in both univariate and multivariate analyses, age, NLR, and CRP values demonstrated significant (*p* < 0.05) predictive influence on hospitalization duration.

**Conclusion:**

Age emerges as a determinant that affects hospital stay duration in DNIs. Moreover, NLR is proven to be comparable to CRP in predicting hospitalization length for these patients. NLR's feasibility as a cost-effective predictive marker, being conveniently derived from routine complete blood count assessments, adds to its clinical significance. This study underscores the potential value of NLR and CRP in informing patient management and care strategies for DNIs.

## Highlights

• The Role of NLR and CRP in hospitalization duration for neck ınfections.
• Retrospective Analysis: 275 Patients (2016–2021), Mean Age 36, Mean Stay 9.6 Days.
• Age, NLR, and CRP as predictors of hospitalization duration in neck ınfections.
• Significance of Age in prognosticating hospital stay duration for neck ınfections.
• NLR as a Cost-Effective and clinically significant marker in neck ınfections.

## Introduction

Deep Neck Infections (DNIs) refer to suppurative infections that develop within the neck's potential spaces and fascial planes.^1^ Although the mortality rates associated with DNIs were notably high before the advent of antibiotics, they still remain a significant threat to life.^2^ Current estimates suggest that the direct economic burden of DNIs surpasses $200 million annually in the United States.^3^ A comprehensive analysis of pediatric patients in the United States revealed an incidence rate of 4.6 cases per 100,000 individuals, with an annual cost exceeding $75 million.^4^

In the treatment process of patients with DNI, securing the airway is paramount. Severe upper airway obstruction is a common and dangerous complication that necessitates prompt intervention. Tracheotomy plays a critical role in managing these cases by ensuring a secure airway and preventing life-threatening respiratory distress. In addition to airway management, the length of hospital stay is a crucial criterion for the efficient use of health resources.

A study exploring the determinants of hospital stay duration identified several prognostic indicators. These included the presence of comorbidities, involvement of non-odontogenic sources, leukocyte count exceeding 11.0 cells × 10^9^/L upon admission, and the requirement for both medical and surgical interventions.^5^ Another investigation focusing on factors influencing hospital stay length in DNIs emphasized that age, diabetes, and the need for repetitive surgical procedures were associated with prolonged hospitalization.^6^

In our study, we aim to investigate the effect of Neutrophil/Lymphocyte Ratio (NLR) and C-Reactive Protein (CRP) levels on the duration of hospitalization for DNI patients. By elucidating the relationship between these markers and hospital stay, our research seeks to provide insights that could enhance clinical decision-making, optimize resource allocation, and ultimately improve patient care. Understanding these associations could lead to more personalized treatment approaches, reducing the length of hospital stays and the associated healthcare costs.

## Methods

Our retrospective study included patients of all age groups hospitalized for DNIs between January 2016 and January 2021 at our clinic, conducted at Bakırköy Dr Sadi Konuk Training and Research Hospital's Ear Nose and Throat Diseases Clinic in accordance with the principles of the Declaration of Helsinki and the Good Clinical Practices Guide, with ethical approval obtained from the Bakırköy Dr Sadi Konuk Training and Research Hospital Clinical Research Committee (reference number 2022/285, Annex 2).

The study excluded patients who declined treatment, were lost to follow-up, sought treatment after abscess drainage elsewhere, had acquired immunodeficiency, had smaller abscess sizes in outpatient settings, manageable infections limited to a single site, had prior steroid treatment, had conditions affecting leukocyte counts unrelated to infection, and those who did not provide consent. Patient records assessed demographic data, etiology, comorbidities, imaging findings, prescribed antibiotics, interventional procedures, laboratory results, culture results, hospitalization duration, complications, and fatalities.

DNIs were classified based on specific areas. Radiological and clinical evaluations were performed to determine the localization. Empirical antibiotic therapy was initiated for all patients, and surgical intervention was considered when necessary for airway safety, large abscesses, lack of response to antibiotics, complications, general condition disorders, and insufficient oral intake.

Patients underwent various interventional procedures, including needle aspiration, ultrasound-guided drainage, incision drainage, and surgical interventions. Complications during treatment were documented. Radiological evaluation was performed for all patients, except those with no oral intake issues, no suspected involvement of other areas, and symptom relief following drainage.

Discharge criteria included clinical improvement, normalized hemogram and CRP, absence of abscess, and satisfactory oral intake. Patients discharged before 14 days had extended antibiotic therapy based on infectious disease specialist recommendations. The study evaluated the association between age, neutrophil and lymphocyte counts, NLR, CRP, and length of hospital stay. Patients were divided into two groups: less than 7 days and 7 days or more. Univariate and multivariate analysis of variance were performed to investigate these associations.

### Statistical analysis

For the statistical analysis, Statistical Package for the Social Sciences Armonk, New York (SPSS) 28.0 was used. Descriptive statistics included mean, standard deviation, median, minimum, maximum, frequency, and ratios. Fischer's exact test was used to analyze treatment distribution by localization. The Kruskal-Wallis test was used for length of stay by localization. The Kolmogorov-Smirnov test measured variable distribution. The Mann–Whitney *U* test analyzed quantitative independent data, and the Chi-Square test analyzed qualitative independent data. Receiver Operating Characteristic curve was used to investigate effect level and cut-off value. Univariate and multivariate logistic regression analyzed the effect level. Statistical significance was set at *p* < 0.05.

## Results

Our study involved the identification of 324 patients who were hospitalized in our clinic due to DNIs. Out of these, 49 patients were excluded from the study as they did not meet the inclusion criteria. Ultimately, our analysis included a total of 275 patients who met the criteria and for whom data were available.

The age range of the patients included in the study was 1–89 years, with a mean age of 36 ± 20.2 years. Among the patients, 170 (61.8%) were male and 105 (38.2%) were female. The mean duration of hospital stay for all patients was 9.6 ± 6.6 days, ranging from 1 to 41 days.

The mean neutrophil count was 11,245 ± 5,433 µL, the mean lymphocyte count was 2,142 ± 945 µL, the mean neutrophil/lymphocyte ratio was 7 ± 8.1, and the mean CRP value was 135.9 ± 110.5 mg/L (Table 1).

**Table 1:** Analysis of demographic and laboratory values of the patients included in the study.

	Minimum‒Maximum		Median	Mean ± SD	
Age	1.0	89.0	34.0	36.0	20.2
Length of stay (days)	1.0	41.0	8.0	9.6	6.6
Neutrophil (µL)	230	35300	10470	11245	5433
Lymphocyte (µL)	230	6720	2070	2142	945
NLR	0.3	94.8	4.9	7.0	8.1
CRP	1.7	760.0	102.0	135.9	110.5

Among the entire study group, tonsillopharyngeal infections were the most prevalent, accounting for 95 patients (34%). The source of infection could not be determined in 87 patients (31.6%), and odontogenic infections were detected in 79 patients (28.7%) (Table 2).

**Table 2:** Etiological factors in the whole patient group.

Etiology	n = 275 (%)
Tonsillopharyngeal infection	95 (34.5%)
Source not found	87 (31.6%)
Odontogenic infection	79 (28.7%)
Branchial cleft infection	3 (1.1%)
Foreign body	2 (0.72%)
Sialolithiasis	2 (0.72%)
Parotid	2 (0.72%)
Jaw trauma	1 (0.36%)
Thyroiditis	1 (0.36%)
Parotid surgery	1 (0.36%)
Bee sting	1 (0.36%)
Otitis externa	1 (0.36%)

In terms of medical history, 232 (84.4%) patients had no history of chronic diseases, while 43 (15.6%) patients had at least one chronic condition. The prevalent chronic diseases observed were diabetes in 31 patients (11%). 255 out of 275 patients (92.7%) underwent radiological evaluation.

The study examined the regional involvement and treatment outcomes of DNIs. The peritonsillar area was affected in 93 patients (33.8%), followed by the submandibular area in 88 patients (32%), parapharyngeal area in 30 patients (11%), parotid area in 20 patients (7.3%), retropharyngeal area in 17 patients (6.2%), submental area in 15 patients (5.4%), and anterior visceral area in 12 patients (4.3%).

Treatment approaches varied; with 67 patients (24.3%) receiving medical treatment alone, while 208 patients (75.6%) underwent surgery for abscess drainage. Specific treatments included incision and drainage in 80 patients (29.1%), needle aspiration in 61 patients (22.2%), and surgical exploration in 54 patients (19.6%).

Microbiological examination was not conducted in patients who received medical treatment alone, and culture studies couldn’t be performed in 53 patients (25.4%) who underwent intervention. Among the patients who underwent culture studies (155 patients), no growth was observed in the culture material of 79 patients (37.9%), while reproduction was achieved in the remaining 76 patients (36.5%).

During the study, complications were observed in 14 patients (5.1%). Severe upper airway obstruction occurred in 8 patients, necessitating tracheotomy to secure the airway. Mediastinitis was observed in 3 patients (1.1%), sepsis in 2 patients (0.7%), and disseminated intravascular coagulation in 1 patient (0.4%). Additionally, 3 patients (1.1%) died because of DNIs.

When the length of stay is evaluated according to the localization, peritonsillar area infections had the shortest hospital stay with mean of 5.5 ± 3.34 days, while the longest mean hospital stay was recorded for retropharyngeal area infections with 17.56 ± 9.57 days.

When the groups with hospitalization duration ≤7 days and >7 days were compared, no significant difference (*p* = 0.773) was observed between two genders. Patient’s age (*p* < 0.001), neutrophil value (*p* = 0.024), NLR (*p* = 0.001) and CRP value (*p* = 0.001) were significantly higher in the group with hospitalization duration >7 days than in the group with hospitalization duration ≤7 days. The lymphocyte value was significantly lower in the group with hospitalization duration >7 days than in the group with hospitalization duration ≤7 days (*p* = 0.002). Analysis of demographic and laboratory data according to length of stay is shown in Table 3.

**Table 3:** Analysis of demographic and laboratory data according to length of stay.

	Length of stay ≤7 days			Length of stay >7 days			*p*
	Mean ± SD	n (%)	Median	Mean ± SD	n (%)	Median	
Age	30.4 ± 18.2		27.0	41.3 ± 20.7		40.0	**0.000**^a^
Gender							
Female		50 (37.3%)			55 (39.0%)		0.773^b^
Male		84 (62.7%)			86 (61.0%)		
Length of stay (days)	5 ± 1.6		5.0	13.9 ± 6.6		12.0	**0.000**^a^
Neutrophil (µL)	10302 ± 4599		9900	12142 ± 6000		10730	**0.024**^a^
Lymphocyte (µL)	2294 ± 959		2270	1998 ± 912		1890	**0.002**^a^
NLR	5.3 ± 3.6		4.3	8.6 ± 10.5		5.6	**0.001**^a^
CRP	110.5 ± 88.4		85.0	160.1 ± 123.5		144.0	**0.001**^a^

SD, Standart Deviation.

a Mann–Whitney µ test.

b Chi-Square test.

Age (*p* < 0.001) [Area under the curve 0.661 (0.597‒0.726)], neutrophil value (*p* = 0.024) [Area under the curve 0.579 (0.512‒0.646)], lymphocyte value (*p* = 0.02) [Area under the curve 0.609 (0.542‒0.676)], NLR value (*p* = 0.001) [Area under the curve 0.619 (0.553‒0.685)], CRP value (*p* = 0.001) [Area under the curve 0.616 (0.549‒0.682)] are found to be significantly effective in terms of length of hospital stay. The area under the curve and the confidence interval of demographic and laboratory data are shown in Table 4.

**Table 4:** The area under the curve and confidence interval of demographic and laboratory data.

	Area under the curve	95% Confidence interval	*p*
Age	0.661	0.597‒0.726	**<0.001**
Neutrophil	0.579	0.512‒0.646	**0.024**
Lymphocyte	0.609	0.542‒0.676	**0.002**
NLR	0.619	0.553‒0.685	**0.001**
CRP	0.616	0.549‒0.682	**0.001**

In the univariate model, age (*p* < 0.001), neutrophil (*p* = 0.006), NLR (*p* = 0.001) and CRP values (*p* < 0.001) were significantly higher, while lymphocyte value (*p* = 0.011) was significantly lower in predicting patients with a hospitalization period of less than or over 7 days (Table 5).

**Table 5:** Analysis of age and laboratory data in a univariate and multivariate model.

	Univariate model			Multivariate model		
	OR	95% CI	*p*	OR	95% CI	*p*
Age	1.029	1.016‒1.042	**<0.001**	1.026	1.012‒1.039	**<0.001**
Neutrophil	1.000	1.000‒1.000	**0.006**			
Lymphocyte	1.000	0.999‒1.000	**0.011**			
NLR	1.107	1.045‒1.173	**0.001**	1.064	1.051‒1.135	**0.041**
CRP	1.004	1.002‒1.007	**<0.001**	1.003	1.001‒1.005	**0.048**

Logistic Regression (Forward LR). CI, Confidence Intervals; OR, Odds Ratio.

In the multivariate reduced model, significant independent efficacy of high age, NLR and CRP values was observed in predicting patients with a hospital stay of less than or more than 7 days (*p* < 0.001, *p* = 0.041, *p* = 0.048) (Table 5).

**Fig. 1:**
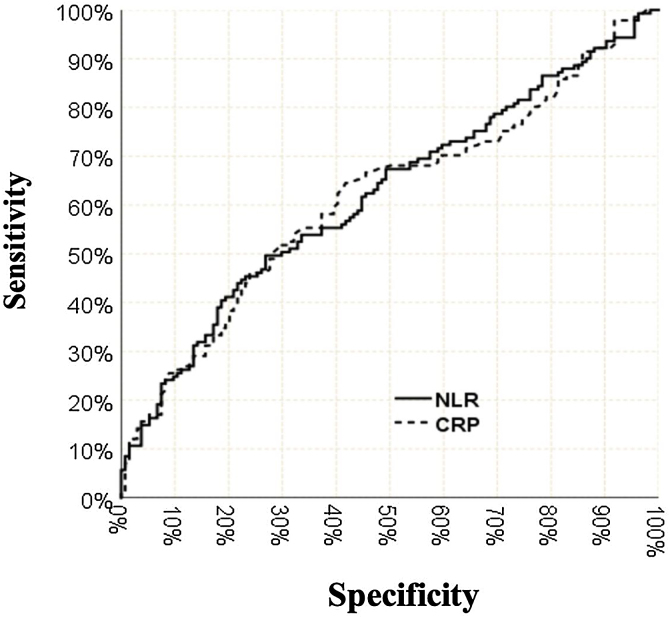
ROC curve of NLR and CRP.

Sensitivity was 44.7% for the NLR cut-off value of 6.4, the positive predictive value was 67.7%, the specificity was 77.6%, the negative predictive value was 57.1%. Sensitivity was 46.1% for the CRP cut-off value of 156 mg/L, the positive predictive value was 67%, specificity 76.1%, negative predictive value was 57.3% (Table 6). The ROC curve of NLR, CRP is shown in Fig. 1.

**Table 6:** Analysis of cut-off values of NLR and CRP.

		Length of stay		Sensitivity	PPV	Specificity	NPV
		≤7 Days	>7 Days				
NLR	≤6.4	104	78	44.7%	67.7%	77.6%	57.1%
	>6.4	30	63				
CRP	≤156	102	76	46.1%	67.0%	76.1%	57.3%
	>156	32	65				

PPV, Positive Predictive Value; NPV, Negative Predictive Value.

## Discussion

The mean age and female to male ratio of our patients hospitalized for DNI were similar to those in the literature.^7^, ^8^, ^9^, ^10^, ^11^, ^12^, ^13^, ^14^ Consistent with the literature, diabetes is the most common comorbidity accompanying DNIs.^11^, ^12^, ^14^, ^15^, ^16^, ^17^, ^18^, ^19^, ^20^, ^21^ Alike the previous research, tonsillopharyngeal and odontogenic infections are the two most common etiological factors that can be identified.^7^, ^9^, ^11^, ^13^^,^^14^, ^16^, ^22^, ^23^ Since peritonsillar region infections were also included in our study, this was the region with the highest involvement.^10^, ^13^, ^18^, ^24^ In studies that did not include infections in this region, the submandibular region was the most frequently involved; It is the second most frequently involved region in our study.^11^, ^16^, ^17^, ^23^

The rates of patients who receive only medical treatment in DNIs differ in the literature.^7^, ^17^, ^23^, ^24^, ^25^ Variations in hospitalization criteria, surgical intervention protocols, patient demographics, and the infected areas in those studies may account for the differences.

The most commonly preferred antibiotics for DNI treatment include amoxicillin-clavunate, ampicillin-sulbactam, second/third-generation cephalosporins, metronidazole, and clindamycin, either alone or in combination.^7^, ^12^, ^13^, ^17^^,^^18^, ^23^, ^24^, ^25^, ^26^, ^27^ In our study, combination therapy involving third-generation cephalosporins and either clindamycin or metronidazole was the predominant approach.

In addition to medical treatment, invasive procedures may be necessary for some patients. The specific procedures applied varied, such as transoral-cervical-combined approach, emergency or elective interventions, needle drainage, incision drainage, exploration, and administration of local or general anesthesia. In our study, the most frequently performed invasive intervention was incision and drainage.^9^, ^11^, ^13^, ^17^^,^^19^, ^23^, ^24^, ^28^

Streptococci are frequently identified as the most prevalent bacterial species in the literature, with the most common subgroups being Streptococcus pyogenes and Viridans group Streptococci.^12^, ^13^, ^15^, ^19^^,^^22^, ^24^, ^29^, ^30^, ^31^ Similarly in our study, Streptococci were the most frequently detected species.

One of the critical factors influencing the course of DNI is the intensity of the infection and the extent to which the patient is at risk, particularly regarding airway compromise. Upper airway obstruction in DNIs is the most common complication reported in the literature.^15^, ^17^, ^18^, ^21^^,^^23^ Severe infections can cause significant swelling and abscess formation, potentially obstructing the airway and necessitating urgent medical attention. In patients presenting with symptoms of airway obstruction, such as difficulty breathing, stridor, or severe dysphagia, intubation or, in life-threatening situations, tracheotomy may be required to secure the airway. The consensus across all sources is that securing the patient's airway without hesitation is crucial in managing this complication. Therefore, performing a tracheotomy in patients with upper airway obstruction is lifesaving. In our study, upper airway obstruction was the most common complication, and our complication and mortality rates were similar to those reported in the literature.^11^, ^16^, ^18^, ^32^^,^^33^ We performed tracheotomies to secure the airway in patients with upper airway obstruction.

In the literature, hospital stays for DNIs vary from 4.7 to 13 days.^7^, ^11^, ^12^, ^13^, ^16^, ^17^, ^24^^,^^28^, ^34^ Our study observed a mean hospital stay of 9.6 ± 6.6 days, consistent with previous findings. Differences in discharge criteria and patient populations contribute to the variability in hospitalization duration across studies. O'Brien et al. identified age, diabetes, and repeated surgical procedures as factors influencing the length of hospital stay in DNIs.^6^ In our study, multivariate analysis revealed a significant and independent association between age and hospitalization duration (*p* < 0.001), with elderly patients experiencing longer stays. In both Gorjon's study and our study, the shortest hospitalization period was observed in peritonsillar space infections.^18^

A Complete Blood Count (CBC) is a necessary laboratory test for almost every patient with a DNI. It is a valuable and relatively inexpensive diagnostic tool. Additionally, NLR can be derived from CBC. NLR is a marker of systemic inflammation. During an infection, the body increases neutrophil production to combat pathogens, while lymphocyte production might decrease due to redistribution or suppression.^35^ An elevated NLR reflects a heightened inflammatory response.^36^ In deep neck infections, a higher NLR could indicate more severe or extensive infection.^36^, ^37^

Recent investigations have explored the prognostic value of NLR as a marker of systemic inflammation in various diseases, including head and neck cancers, colorectal cancer, non-small cell lung cancer, stomach cancer, prostate cancer, ST-elevation myocardial infarction, peripheral vertigo, psoriasis, diabetic peripheral neuropathy, psoriasis, Helicobacter pylori-related gastritis, and complicated appendicitis.^38^, ^39^, ^40^, ^41^, ^42^, ^43^, ^44^

CRP is a non-specific acute phase protein produced by the liver in response to pro-inflammatory cytokines like IL-6.^45^ It rises rapidly within hours of inflammation onset and has a short half-life, making it useful for monitoring acute inflammatory responses.^45^ High CRP levels in deep neck infections suggest significant inflammation, correlating with infection severity and leading to prolonged hospitalization for intensive treatment and recovery.^46^

Understanding the mechanisms underlying the relationship between NLR, CRP, and hospitalization duration in deep neck infections underscores the importance of these markers in clinical practice. Elevated NLR and CRP levels indicate infection severity and serve as valuable tools for monitoring treatment response and predicting patient outcomes. These biomarkers can guide clinical decision-making, helping to tailor treatment plans and potentially improve patient prognosis by identifying those at risk for longer hospital stays and complications.

Ban et al. investigated the markers that determine the success of surgical drainage in DNIs and identified CRP and NLR among them. They established estimated cut-off values for CRP and NLR at 41.25 mg/L and 8.02, respectively. Consequently, NLR emerged as a marker that can predict drainable abscesses.^47^ CRP also appears as a prognostic marker in DNIs, specifically related to the length of hospital stay.^46^, ^48^, ^49^, ^50^ In Liu et al.'s study, they reported that persistent and continuous discharge after incision drainage in patients with DNIs was independently associated with age over 55, preoperative CRP levels exceeding 15 mg/dL, and preoperative blood glucose levels surpassing 8.3 mmol/L.^51^

Multiple studies in the literature have examined the association between NLR and DNI.^8^, ^36^, ^37^, ^47^^,^^52^, ^53^ Baglam et al. investigated NLR as a supportive marker for diagnosing DNI in patients with acute bacterial tonsillitis. They found significantly higher NLR values in patients with acute bacterial tonsillitis and DNI, with NLR showing high sensitivity and specificity for predicting deep neck abscesses.^52^ Gallagher et al. assessed the prognostic value of NLR in odontogenic DNIs and its correlation with CRP levels and hospital stay. They discovered a significant relationship between CRP, NLR, and length of hospital stay, with identified cut-off values for NLR and CRP in patients with a hospital stay of two or more days.^8^

The objective of our study was to assess the clinical utility of NLR as a prognostic marker in DNI and its relationship with CRP levels and length of hospital stay. In the multivariate analysis, NLR and CRP showed significant independent predictive value in determining patients with a hospital stay of less than 7 days (*p* = 0.041, *p* = 0.048, respectively). Using a NLR cut-off value of 6.4, the sensitivity, specificity, positive predictive value, and negative predictive value were determined as 44.7%, 77.6%, 67.7%, and 57.1%, respectively. For CRP’s cut-off value of 156 mg/L, the corresponding values were 46.1%, 76.1%, 67.0%, and 57.3%. Our findings suggest that NLR is comparable to CRP in predicting the length of hospital stay in patients diagnosed with DNI. Furthermore, NLR is a cost-effective prognostic marker that can be easily calculated from CBC. The cut-off values determined for NLR and CRP in our study align with previous research.^8^, ^47^, ^48^, ^49^, ^51^, ^52^

A major limitation of our study is its reliance on retrospective analysis from a single center, potentially limiting the generalizability of the findings. Additionally, although our clinic follows a standardized treatment protocol, there may be variations in the approach among different physicians performing surgical procedures. The study excluded patients with conditions affecting leukocyte counts unrelated to infection parameters: those receiving prior steroid treatment, and individuals with acquired immunodeficiency. This exclusion might limit the applicability of the findings to these specific populations.

Although multivariate analyses were performed, there may still be unmeasured confounders that could influence the association between NLR, CRP, and hospitalization duration. Factors such as nutritional status, prior antibiotic use, and genetic predispositions were not accounted for.

Future research should aim to address these limitations by conducting prospective, multicenter studies with larger sample sizes and standardized treatment protocols. Additionally, including long-term follow-up data and a broader patient population would provide a more comprehensive understanding of the factors influencing hospitalization duration in DNIs.

## Conclusion

The length of hospital stay is an important parameter in patients with DNI, and there is a need for practical methods to predict the length of stay. Cut-off values of NLR > 6.4 and CRP > 156 mg/L were found to be effective in predicting patients whose hospitalization period may be longer than 7 days. NLR was thought to be as effective as CRP in predicting the length of stay and a cost-effective predictor that could be easily calculated from the CBC.

Future research should focus on validating these findings in larger, multicenter cohorts and exploring the integration of NLR into clinical decision-making algorithms. Investigating the potential benefits of early interventions based on NLR and CRP values could enhance patient outcomes and optimize hospital resource utilization. Implementing these predictive markers into everyday clinical practice may help clinicians manage DNI patients more effectively, reducing hospital stay durations and associated healthcare costs.

## Consent to participate

Informed consent was obtained from all individual participants included in the study.

Informed consent was obtained from legal guardians.

## Consent to publish

Parents signed informed consent regarding publishing their data and photographs individual participants signed informed consent regarding publishing their data and photographs.

## Ethics approval

Our study was conducted at Bakırköy Dr Sadi Konuk Training and Research Hospital's Department of Otorhinolaryngology & Head and Neck Surgery, adhering to the principles of the Declaration of Helsinki and Good Clinical Practices Guide. Ethical approval was obtained from the Bakırköy Dr Sadi Konuk Training and Research Hospital Clinical Research Committee (reference number 2022/285, Annex 2).

## Funding

All authors certify that they have no affiliations with or involvement in any organization or entity with any financial interest or non-financial interest in the subject matter or materials discussed in this manuscript.

## Conflicts of interest

The authors declare that they have no known competing financial or non-financial interests or personal relationships that could have appeared to influence the work reported in this paper.
